# Temporal and spatial trends of accidents with venomous animal in Brazil before and during the COVID-19 pandemic: a population-based ecological study

**DOI:** 10.1590/1980-549720250012

**Published:** 2025-04-07

**Authors:** Thayane Santos Siqueira, Lívia Silveira Silva, Jamile Rodrigues Cosme de Holanda, Sálvia Cely Cerqueira Carvalho, Adriano José dos Santos, Alexrangel Henrique Cruz Santos, José Rodrigo Santos Silva, Victor Santana Santos

**Affiliations:** I Universidade Federal de Sergipe, Postgraduate Program Stricto Sensu in Health Sciences - Aracaju (SE), Brazil.; II Instituto Osvaldo Cruz, Postgraduate Program Stricto Sensu in Vector Malacology - Rio de Janeiro (RJ), Brazil.; III Universidade Federal de Sergipe, Postgraduate Program Stricto Sensu in Parasitic Biology - São Cristóvão (SE), Brazil.; IV Universidade Federal de Sergipe, Department of Statistics and Actuarial Sciences - Aracaju (SE), Brazil.; V Universidade Federal de Sergipe, Postgraduate Program Stricto Sensu in Sciences Applied to Health - Lagarto (SE), Brazil.; VI Universidade Federal de Sergipe, Department of Medicine - Lagarto (SE), Brazil.

**Keywords:** Venomous animals, Geospatial, Health information systems, COVID-19, Epidemiology, Public health, Animais peçonhentos, Geoespacial, Sistemas de informação em saúde, COVID-19, Epidemiologia, Saúde pública

## Abstract

**Objective:**

The objective of this study was to analyze the temporal and spatial trends of accidents involving venomous animals in Brazil during the pre- and COVID-19 pandemic periods.

**Methods:**

We conducted a population-based ecological study using comprehensive data from the Notifiable Diseases Information System, covering all accidents involving venomous animals in Brazil from January 2013 to December 2022. We did a temporospatial analysis to compare the incidence rates of accidents involving venomous animals per Brazilian municipality in the pre-pandemic period (January 2013 to February 2020) and the pandemic period (March 2020 to December 2022). To analyze the trend, the seasonal-trend model was used based on the classic additive decomposition model. For spatial distribution analysis, the Global Moran’s Index was used.

**Results:**

A total of 2,202,842 cases of accidents involving venomous animals were recorded. Brazil showed an increasing trend from 2017 to 2019 (annual percentage change [APC]: 0.98, p<0.001) and a stable trend from 2020 to 2022 (APC: 0.42, p<0.080). The North (APC: 0.19, p<0.330), South (APC: 0.04, p<0.953), and Southeast (APC: 0.26, p<0.312) regions presented a stable trend from 2020 to 2022. Spatial dependence of smoothed rates was observed in both the pre-pandemic (Moran’s I: 0.47; p=0.000) and COVID-19 pandemic periods (Moran’s I: 0.50; p=0.000).

**Conclusion:**

There was a stable trend in accidents involving venomous animals from 2020 to 2022 in Brazil. The spatial distribution of cases was heterogeneous for both periods studied.

## RESUMO

**Objetivo:**

Analisar as tendências temporais e espaciais dos acidentes envolvendo animais peçonhentos no Brasil nos períodos pré e pós-pandemia.

**Métodos:**

Realizamos um estudo ecológico de base populacional, no qual foram coletados dados do Sistema de Informação de Agravos de Notificação sobre todos os acidentes envolvendo animais peçonhentos no Brasil no período de janeiro de 2013 a dezembro de 2022. Calculamos as taxas de incidência de acidentes com animais peçonhentos por município brasileiro e comparamos temporalmente e espacialmente o período pré-pandemia (janeiro de 2013 a fevereiro de 2020) com o período pós-pandemia (março de 2020 a dezembro de 2022). Para analisar a tendência, foi utilizado o modelo de tendência sazonal baseado no método clássico de decomposição aditiva. Para a análise da distribuição espacial foi utilizado o Índice Global de Moran.

**Resultados:**

Foram registrados 2.202.842 casos de acidentes envolvendo animais peçonhentos. O Brasil apresentou tendência crescente de 2017 a 2019 (*annual percentage* change - APC 0,98, p<0,001) e tendência estável de 2020 a 2022 (APC 0,42, p<0,080). As Regiões Norte (APC: 0,19, p<0,330), Sul (APC: 0,04, p<0,953) e Sudeste (APC: 0,26, p<0,312) apresentaram tendência estável de 2020 a 2022. A dependência espacial das taxas suavizadas foi observada tanto pré (Moran I: 0,47; p=0,000) quanto pós-pandemia de COVID-19 (Moran I: 0,50; p=0,000).

**Conclusão:**

Houve tendência estável de acidentes envolvendo animais peçonhentos de 2020 a 2022 no Brasil. A distribuição espacial dos casos foi heterogênea nos dois períodos estudados.

## INTRODUCTION

Accidents involving venomous animals are a significant public health issue, especially in tropical countries^1^. Due to the high frequency of venomous animals and their wide potential to cause accidents, with associated morbidity, especially in populations with high social vulnerability, the World Health Organization (WHO) has included accidents involving venomous animals in the list of neglected tropical diseases (NTDs)^1^ ^-^ ^3^.

In Brazil, accidents involving these types of animals are compulsorily notifiable^2^. Within the diversity of the Brazilian fauna and flora, some animals stand out with respect to the occurrence and severity of accidents caused by venomous animals, particularly scorpions, spiders, snakes, bees, and caterpillars^4^ ^,^ ^5^. However, the incidence of accidents caused by venomous animals may vary over time and location according to variables related to anthropic action, climatic changes, disorderly urban growth, and changes in the food chain, among others^5^ ^,^ ^6^.

Additionally, the COVID-19 pandemic may have directly influenced changes in the incidence of accidents involving venomous animals in Brazil. Emergency measures implemented by governments and local authorities to mitigate the spread of severe acute respiratory syndrome-coronavirus-2 (SARS-CoV-2) and prevent strain on healthcare systems included reallocating healthcare personnel, suspending non-urgent medical services, enhancing COVID-19 information systems, and promoting social isolation^7^ ^,^ ^8^.

Therefore, it is necessary to understand whether there has been a change in the spatiotemporal dynamics of accidents involving venomous animals. Herein, we conducted a comprehensive analysis to investigate the spatiotemporal distribution of accidents caused by venomous animals in Brazil during the pre- and COVID-19 pandemic periods from January 2013 to December 2022.

## METHODS

### Study design

This ecological analytical population-based study investigated the temporal trends and spatial distribution of all notified cases of venomous animal accidents from January 2013 to December 2022. We compared temporally and spatially the incidence rates of accidents with venomous animal per Brazilian municipality in the pre-pandemic period (January 2013 to February 2020) and the pandemic period (March 2020 to December 2022). The units of analysis were the 5,572 municipalities of Brazil. All analyses considered the municipalities where the accidents had occurred.

### Study area

**Figure 1:**
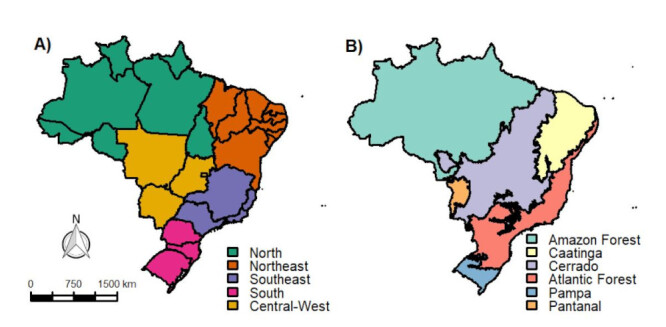
(A) Division of Brazilian regions: North, Northeast, Southeast, South, and Central-West. (B) Biomes of Brazil: Amazon Forest, Caatinga, Cerrado, Atlantic Forest, Pampas, and Pantanal.

Brazil is a continental-sized country located in South America, with a population of approximately 210 million inhabitants, ranking sixth in population and fifth in territorial extension worldwide, covering an area of approximately 8.51 million km^2^, with 22.43 inhabitants per square kilometer^9^. Administratively, it is composed of 5,572 municipalities, which make up its 27 states, and the Federal District of Brasília. Brazil is divided into five regions (North, Northeast, Central-West, Southeast, and South) (Figure 1A), with significant differences in population density and socioeconomic aspects between them-Subtropical, Temperate, Semiarid, and Arid, with each of them having different types of vegetation and fauna (Figure 1B)^10^.

### Data source and measures

The number of cases of accidents with venomous animals was obtained from the Notifiable Diseases Information System (SINAN)^2^. The study period was delimited from 2013 to 2022. We used the annual estimates of the total population by the Brazilian region from the time series to calculate the incidence rate of venomous animal accidents. Demographic data for each municipality were extracted from the 2022 Brazilian Census^9^.

### Data analysis

#### Temporal trend

Trend estimates were conducted using the seasonal trend method (STM), which is based on the classic additive decomposition model and follows the model of detecting breaks in the seasonal and trend components of a univariate time series^11^ ^,^ ^12^. Linear and harmonized terms were fitted to the original time series using ordinary least squares regression, allowing for the detection of trends and trend changes (breakpoints) in a time series^13^. This analysis method consists of a segmented linear regression, using dummy variables, to identify points where there is a change in the trend and estimate the annual percentage change (APC) and the average annual percentage change (AAPC) considering the entire period of the series with a 95% confidence interval (95%CI)^13^.

### Spatial analysis

In the spatial analysis, the incidence coefficients per 100,000 inhabitants were mapped using the cartographic database of Brazil, divided by municipalities, available on the IBGE website (https://portaldemapas.ibge.gov.br). The K-means clustering technique^14^, with the Hartigan-Wong algorithm, was used for municipality stratification. Crude rates were smoothed using the Local Empirical Bayesian Estimator to minimize instability caused by random fluctuations^15^. The Global Moran’s Index^16^ was calculated to identify spatial autocorrelations, and, when identified, the Local Indicator of Spatial Association (LISA) was used to quantify the degree of spatial association to which each location in the sample set was subjected based on a neighborhood model. LISA results in the Moran scatter plot were used to identify critical or transition areas. The generated quadrants were interpreted as follows: Q1 - High/High (positive values and positive means); Q2 - Low/Low (negative values and negative means); Q3 - High/Low (positive values and negative means); Q4 - Low/High (negative values and positive means). LISA maps consider only areas whose indices are significant. We employed flexible spatial scan statistics with a Poisson probability model using a likelihood log ratio and 10 census tracts as the maximum size of the spatial cluster to identify clusters of venomous animal accidents and estimate relative risks (RRs).

### Software used

Data processing, organization, and all statistical analyses, including temporal analysis and spatial analysis, were performed using the R software, version 4.3.2 (The R Core Team, 2023). The significance level adopted was 5%. The download and manipulation of the shapefile files were done using the “geobr,” “sf,” and “sp” packages. For the classification of the incidences, the K-means was applied with the help of the “vegan” and “classint” packages. For the Global Moran’s Index and LISA, the “spdep,” “prioritizr,” “ape,” and “rgeoda” packages were used. The spatial scan analysis was performed with the help of the “rflexscan” package. 

### Ethical considerations

Institutional review board approval and informed consent were not required, as all data were obtained from publicly available databases.

## RESULTS

**Figure 2:**
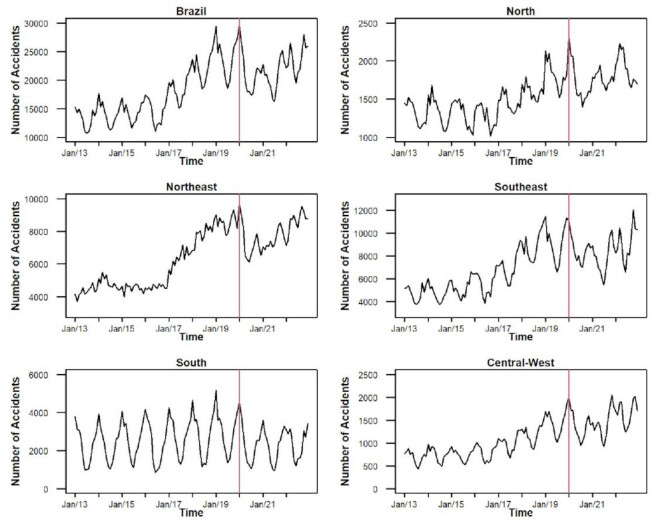
Temporal evolution of the incidence rates of accidents with venomous animals in Brazil and in the five Brazilian regions from 2013 to 2022.

From 2013 to 2022, a total of 2,202,842 cases of accidents involving venomous animals were reported in Brazil. Scorpion stings (58.40%) had the highest rate, followed by spider bites (14.31%), snakebites (13.09%), bee stings (7.88%), others (4.24%), and caterpillar stings (2.08%). Scorpion stings had the highest rate in the Southeast region in both the pre-pandemic period (153.13/100,000 inhabitants) and the pandemic period (237.54/100,000 inhabitants), and in the Northeast region during the pre-pandemic period (80.93/100,000 inhabitants) and the pandemic period (118.42/100,000 inhabitants). Snakebites predominated in the North region in the pre-pandemic (74.65/100,000 inhabitants) and pandemic periods (80.27/100,000 inhabitants). Spider bites had the highest rate in the South region in both the pre-pandemic (113.52/100,000 inhabitants) and COVID-19 pandemic periods (87.67/100,000 inhabitants) (Figure 2 and Supplementary Tables 1 and 2).

**Supplementary Table 1:** Temporal analysis of incidence rates according to the type of venomous animal accident in Brazil and its five regions between 2013 and 2022.

Variable	2013 to 2016				2017/2013* to 2019				2020/2013** to 2022			
	APC	95%CI	Trends	P-value	APC	95% CI	Trends	P-value	APC	95%CI	Trends	P-value
Brazil	0.16	-0.13 to 0.45	Stable	0.273	0.98	0.55 to 1.40	Increasing	<0.001	0.42	-0.05 to 0.89	Stable	0.080
Snake*					0.15	-0.05 to 0.36	Stable	0.135	-0.77	-1.52 to -0.03	Decreasing	0.043
Spider*					0.39	0.15 to 0.64	Increasing	0.002	-0.09	-0.98 to 0.81	Stable	0.842
Scorpion	0.46	0.25 to 0.67	Increasing	<0.001	1.42	1.05 to 1.79	Increasing	<0.001	0.63	0.22 to 1.05	Increasing	0.004
Caterpillar**									0.16	-0.25 to 0.57	Stable	0.434
Bee	0.21	-0.36 to 0.79	Stable	0.461	1.20	0.54 to 1.87	Increasing	0.001	1.14	0.28 to 2.01	Increasing	0.011
Others	0.18	-0.29 to 0.65	Stable	0.438	0.78	0.15 to 1.41	Increasing	0.017	0.90	0.15 to 1.65	Increasing	0.020
North	-0.28	-0.54 to -0.02	Decreasing	0.036	0.68	0.35 to 1.01	Increasing	<0.001	0.19	-0.20 to 0.58	Stable	0.330
Snake	-0.50	-0.89 to -0.11	Decreasing	0.012	0.40	-0.20 to 1.00	Stable	0.183	-0.52	-1.13 to 0.08	Stable	0.089
Spider	-0.46	-0.89 to -0.03	Decreasing	0.036	0.25	-0.37 to 0.87	Stable	0.418	0.72	-0.13 to 1.57	Stable	0.093
Scorpion	-0.29	-0.56 to -0.02	Decreasing	0.034	1.12	0.75 to 1.49	Increasing	<0.001	0.76	0.33 to 1.18	Increasing	0.001
Caterpillar	-2.89	-4.35 to -1.43	Decreasing	<0.001	-0.15	-2.52 to 2.22	Stable	0.900	-0.76	-3.05 to 1.53	Decreasing	0.504
Bee	0.14	-0.32 to 0.60	Stable	0.537	1.68	1.06 to 2.31	Increasing	<0.001	1.33	0.45 to 2.20	Increasing	0.004
Others**									0.85	0.73 to 0.97	Increasing	<0.001
Northeast	0.09	-0.05 to 0.24	Stable	0.202	1.25	1.02 to 1.47	Increasing	<0.001	0.65	0.30 to 1.00	Increasing	0.001
Snake**									0.40	0.29 to 0.51	Increasing	<0.001
Spider	0.07	-0.25 to 0.38	Stable	0.681	0.96	0.59 to 1.34	Increasing	<0.001	0.91	0.31 to 1.50	Increasing	0.004
Scorpion	0.01	-0.16 to 0.18	Stable	0.888	1.20	0.94 to 1.45	Increasing	<0.001	0.60	0.24 to 0.96	Increasing	0.002
Caterpillar**									0.91	0.61 to 1.21	Increasing	<0.001
Bee	0.61	0.05 to 1.18	Increasing	0.035	2.87	2.27 to 3.46	Increasing	<0.001	1.88	1.18 to 2.58	Increasing	<0.001
Others	0.59	0.31 to 0.87	Increasing	<0.001	1.57	1.09 to 2.04	Increasing	<0.001	1.90	1.14 to 2.65	Increasing	<0.001
Southeast	0.42	0.08 to 0.76	Increasing	0.016	1.10	0.56 to 1.65	Increasing	<0.001	0.26	-0.28 to 0.85	Stable	0.312
Snake**									-0.12	-0.32 to 0.08	Stable	0.228
Spider	-0.22	-0.77 to 0.34	Stable	0.437	0.71	-0.08 to 1.49	Stable	0.076	-0.26	-1.18 to 0.67	Stable	0.580
Scorpion*					1.37	1.22 to 1.51	Increasing	<0.001	0.51	-0.04 to 1.07	Stable	0.070
Caterpillar**									0.17	-0.44 to 077	Stable	0.581
Bee*					0.68	0.40 to 0.96	Increasing	<0.001	0.59	-0.53 to 1.72	Stable	0.293
Others**									0.19	0.00 to 0.39	Increasing	0.047
South					-0.34	-0.07 to 0.75	Stable	0.100	0.04	-1.34 to 1.42	Stable	0.953
Snake**									-0.17	-0.54 to 0.20		0.358
Spider**									-0.16	-0.36 to 0.04		0.117
Scorpion**									1.16	1.02 to 1.50	Increasing	<0.001
Caterpillar**									-0.28	-0.77 to 0.22		0.274
Bee**									0.18	-0.15 to 0.50		0.285
Others**									0.01	-0.32 to 0.34		0.961
Central-West	0.19	-0.25 to 0.63	Stable	0.392	1.39	0.78 to 2.00	Increasing	<0.001	0.71	0.03 to 1.39	Increasing	0.041
Snake**									-0.05	-0.23 to 0.13	Stable	0.570
Spider	-0.20	-0.81 to 0.42	Stable	0.521	0.51	-0.32 to 1.34	Stable	0.219	0.42	-0.54 to 1.39	Stable	0.380
Scorpion	0.45	0.12 to 0.78	Increasing	0.008	2.13	1.63 to 2.62		<0.001	1.11	0.47 to 1.75	Increasing	0.001
Caterpillar**									0.51	-0.05 to 1.06	Stable	0.072
Bee	0.42	-0.28 to 1.12	Stable	0.235	1.76	0.74 to 2.77	Increasing	0.001	1.82	0.84 tp 2.80	Increasing	0.001
Others	0.15	-0.55 to 0.85	Stable	0.668	2.04	1.11 to 2.97	Increasing	<0.001	0.65	-0.34 to 1.65	Stable	0.191

**Supplementary Table 2:** Sociodemographic characteristics of accidents caused by venomous animals in Brazil, by region and study period

Type of Accident	Variable/Category	2013-2019					2020-2022				
		North	Northeast	Southeast	South	Central-West	North	Northeast	Southeast	South	Central-West
Snakes	Age group										
	< 19 years old	19638 (29.9)	12613 (25.2)	7862 (17.6)	3074 (18.6)	4209 (20.9)	8769 (28.2)	6485 (23.4)	2724 (15.9)	998 (16.2)	1607 (19.1)
	20-59 years old	40771 (62.0)	31465 (62.7)	30131 (67.5)	10639 (64.3)	13380 (66.3)	19456 (62.5)	17407 (62.7)	11256 (65.8)	3845 (62.6)	5515 (65.7)
	60 or more	5352 ( 8.1)	6066 (12.1)	6672 (14.9)	2822 (17.1)	2598 (12.9)	2916 ( 9.4)	3880 (14.0)	3132 (18.3)	1302 (21.2)	1276 (15.2)
	Sex										
	Female	13968 (21.2)	12672 (25.3)	10566 (23.7)	4007 (24.2)	4907 (24.3)	6912 (22.2)	6901 (24.9)	4146 (24.2)	1524 (24.8)	2020 (24.1)
	Male	51781 (78.8)	37454 (74.7)	34088 (76.3)	12529 (75.8)	15275 (75.7)	24228 (77.8)	20861 (75.1)	12964 (75.8)	4621 (75.2)	6376 (75.9)
	Race										
	White/Yellow breed	4530 ( 7.2)	4431 (10.3)	19805 (49.0)	13570 (85.3)	5093 (27.9)	1712 ( 5.7)	2089 ( 8.3)	6883 (43.6)	4961 (84.1)	1857 (23.8)
	Indigenous	5161 ( 8.2)	925 ( 2.1)	207 ( 0.5)	241 ( 1.5)	1278 ( 7.0)	2674 ( 8.8)	951 ( 3.8)	92 ( 0.6)	66 ( 1.1)	507 ( 6.5)
	Black/Brown breed	53348 (84.6)	37686 (87.6)	20428 (50.5)	2106 (13.2)	11890 (65.1)	25844 (85.5)	21994 (87.9)	8808 (55.8)	871 (14.8)	5433 (69.7)
Spiders	Age group										
	< 19 years old	1632 (24.2)	3429 (26.3)	14091 (20.9)	27899 (21.3)	1373 (23.5)	855 (21.3)	1834 (24.1)	5886 (19.3)	8687 (19.0)	775 (21.9)
	20-59 years old	4233 (62.9)	8355 (64.1)	41597 (61.8)	81525 (62.2)	3646 (62.5)	2568 (64.0)	4969 (65.2)	18604 (61.1)	28736 (63.0)	2223 (62.8)
	60 or more	865 (12.9)	1251 ( 9.6)	11601 (17.2)	21721 (16.6)	812 (13.9)	588 (14.7)	813 (10.7)	5964 (19.6)	8186 (17.9)	544 (15.4)
	Sex										
	Female	2735 (40.6)	6582 (50.5)	27415 (40.8)	65897 (50.3)	2606 (44.7)	1658 (41.3)	3855 (50.6)	12362 (40.6)	22344 (49.0)	1633 (46.1)
	Male	3995 (59.4)	6452 (49.5)	39854 (59.2)	65239 (49.7)	3225 (55.3)	2352 (58.7)	3761 (49.4)	18088 (59.4)	23261 (51.0)	1909 (53.9)
	Race										
	White/Yellow breed	913 (14.2)	1583 (15.3)	38218 (63.8)	111964 (89.5)	1702 (34.8)	438 (11.3)	831 (12.9)	16477 (59.1)	37527 (87.1)	848 (27.9)
	Indigenous	344 ( 5.3)	62 ( 0.6)	159 ( 0.3)	642 ( 0.5)	91 ( 1.9)	152 ( 3.9)	54 ( 0.8)	79 ( 0.3)	216 ( 0.5)	65 ( 2.1)
	Black/Brown breed	5191 (80.5)	8720 (84.1)	21498 (35.9)	12511 (10.0)	3097 (63.3)	3279 (84.8)	5538 (86.2)	11346 (40.7)	5318 (12.3)	2128 (70.0)
Scorpions	Age group										
	< 19 years old	8125 (27.9)	107607 (29.9)	79141 (22.9)	4300 (21.6)	10100 (24.4)	4714 (26.1)	55046 (27.4)	47921 (21.6)	3115 (20.5)	8397 (24.5)
	20-59 years old	18533 (63.5)	202506 (56.3)	207078 (59.8)	12626 (63.6)	25909 (62.6)	11504 (63.6)	115469 (57.4)	129208 (58.3)	9424 (62.1)	20702 (60.4)
	60 or more	2516 ( 8.6)	49483 (13.8)	59992 (17.3)	2936 (14.8)	5347 (12.9)	1873 (10.4)	30628 (15.2)	44384 (20.0)	2630 (17.3)	5197 (15.2)
	Sex										
	Female	11265 (38.6)	205163 (57.1)	158016 (45.7)	9159 (46.1)	20130 (48.7)	7127 (39.4)	111359 (55.4)	103063 (46.5)	6965 (45.9)	17326 (50.5)
	Male	17903 (61.4)	154366 (42.9)	188098 (54.3)	10701 (53.9)	21222 (51.3)	10964 (60.6)	89734 (44.6)	118383 (53.5)	8201 (54.1)	16961 (49.5)
	Race										
	White/Yellow breed	3782 (13.6)	38630 (14.1)	151488 (47.6)	14365 (76.4)	10713 (33.5)	2025 (11.6)	20286 (12.1)	100492 (48.0)	10543 (72.1)	9118 (32.9)
	Indigenous	728 ( 2.6)	1281 ( 0.5)	1062 ( 0.3)	61 ( 0.3)	315 ( 1.0)	411 ( 2.4)	727 ( 0.4)	785 ( 0.4)	46 ( 0.3)	252 ( 0.9)
	Black/Brown breed	23298 (83.8)	234149 (85.4)	165737 (52.1)	4366 (23.2)	20916 (65.5)	15046 (86.1)	146439 (87.5)	107927 (51.6)	4038 (27.6)	18336 (66.2)
Caterpillars	Age group										
	< 19 years old	869 (47.1)	1437 (37.8)	3790 (29.0)	4042 (33.0)	638 (44.6)	392 (41.4)	758 (36.5)	1681 (25.8)	988 (31.1)	267 (38.5)
	20-59 years old	806 (43.7)	1883 (49.6)	7050 (54.0)	6382 (52.0)	646 (45.1)	452 (47.7)	1040 (50.1)	3601 (55.4)	1679 (52.8)	340 (49.1)
	60 or more	171 ( 9.3)	478 (12.6)	2209 (16.9)	1843 (15.0)	148 (10.3)	103 (10.9)	276 (13.3)	1223 (18.8)	510 (16.1)	86 (12.4)
	Sex										
	Female	866 (46.9)	1725 (45.4)	5844 (44.8)	5541 (45.2)	739 (51.6)	444 (46.9)	955 (46.1)	3005 (46.2)	1503 (47.3)	370 (53.4)
	Male	980 (53.1)	2073 (54.6)	7198 (55.2)	6724 (54.8)	693 (48.4)	503 (53.1)	1117 (53.9)	3496 (53.8)	1674 (52.7)	323 (46.6)
	Race										
	White/Yellow breed	245 (14.2)	436 (14.7)	6932 (60.5)	10608 (90.1)	371 (36.7)	142 (15.6)	237 (13.8)	3288 (55.7)	2696 (88.7)	172 (33.2)
	Indigenous	62 ( 3.6)	20 ( 0.7)	24 ( 0.2)	48 ( 0.4)	22 ( 2.2)	23 ( 2.5)	8 ( 0.5)	11 ( 0.2)	17 ( 0.6)	5 ( 1.0)
	Black/Brown breed	1423 (82.3)	2506 (84.6)	4494 (39.2)	1123 ( 9.5)	617 (61.1)	748 (81.9)	1477 (85.8)	2609 (44.2)	325 (10.7)	341 (65.8)
Bees	Age group										
	< 19 years old	2016 (36.5)	12085 (33.2)	13900 (32.5)	7323 (32.2)	1743 (35.5)	957 (29.5)	7691 (27.4)	4579 (25.8)	2473 (27.5)	814 (25.9)
	20-59 years old	3083 (55.9)	21781 (59.8)	25150 (58.9)	13186 (58.1)	2766 (56.4)	1937 (59.8)	17952 (64.0)	11282 (63.5)	5523 (61.4)	1961 (62.4)
	60 or more	418 ( 7.6)	2579 ( 7.1)	3659 ( 8.6)	2205 ( 9.7)	399 ( 8.1)	346 (10.7)	2396 ( 8.5)	1903 (10.7)	992 (11.0)	366 (11.7)
	Sex										
	Female	1915 (34.7)	13150 (36.1)	15118 (35.4)	8305 (36.6)	1644 (33.5)	1069 (33.0)	9692 (34.6)	5630 (31.7)	3011 (33.5)	947 (30.2)
	Male	3601 (65.3)	23287 (63.9)	27582 (64.6)	14409 (63.4)	3265 (66.5)	2171 (67.0)	18341 (65.4)	12133 (68.3)	5976 (66.5)	2192 (69.8)
	Race										
	White/Yellow breed	808 (15.4)	4367 (15.7)	24262 (64.4)	18601 (85.7)	1306 (34.1)	409 (13.1)	3141 (13.2)	9500 (58.2)	7269 (84.5)	784 (30.0)
	Indigenous	49 ( 0.9)	149 ( 0.5)	90 ( 0.2)	106 ( 0.5)	28 ( 0.7)	24 ( 0.8)	191 ( 0.8)	54 ( 0.3)	29 ( 0.3)	25 ( 1.0)
	Black/Brown breed	4376 (83.6)	23270 (83.7)	13350 (35.4)	2993 (13.8)	2491 (65.1)	2682 (86.1)	20523 (86.0)	6776 (41.5)	1301 (15.1)	1805 (69.1)
Others	Age group										
	< 19 years old	3893 (35.5)	6518 (37.7)	7339 (35.7)	3711 (42.6)	1562 (34.5)	2135 (32.0)	3236 (33.0)	2599 (30.7)	1211 (35.3)	878 (30.2)
	20-59 years old	6404 (58.4)	9207 (53.3)	11343 (55.2)	4175 (48.0)	2512 (55.6)	4011 (60.1)	5583 (56.9)	4927 (58.2)	1764 (51.5)	1715 (59.0)
	60 or more	670 ( 6.1)	1556 ( 9.0)	1859 ( 9.1)	817 ( 9.4)	448 ( 9.9)	524 ( 7.9)	1001 (10.2)	935 (11.1)	451 (13.2)	315 (10.8)
	Sex										
	Female	3513 (32.0)	7883 (45.6)	8903 (43.4)	4126 (47.4)	2073 (45.8)	2304 (34.5)	4464 (45.5)	3560 (42.1)	1617 (47.2)	1429 (49.2)
	Male	7454 (68.0)	9394 (54.4)	11630 (56.6)	4577 (52.6)	2449 (54.2)	4365 (65.5)	5354 (54.5)	4900 (57.9)	1808 (52.8)	1475 (50.8)
	Race										
	Black/Brown breed	1328 (12.9)	1969 (15.0)	10111 (55.4)	7000 (86.7)	1093 (28.5)	726 (11.2)	935 (11.6)	4003 (50.7)	2693 (84.8)	664 (26.4)
	Indigenous	292 ( 2.8)	55 ( 0.4)	40 ( 0.2)	36 ( 0.4)	47 ( 1.2)	151 ( 2.3)	59 ( 0.7)	21 ( 0.3)	13 ( 0.4)	24 ( 1.0)
	Black/Brown breed	8694 (84.3)	11103 (84.6)	8100 (44.4)	1038 (12.9)	2700 (70.3)	5598 (86.5)	7056 (87.7)	3868 (49.0)	469 (14.8)	1827 (72.6)

**Figure 3:**
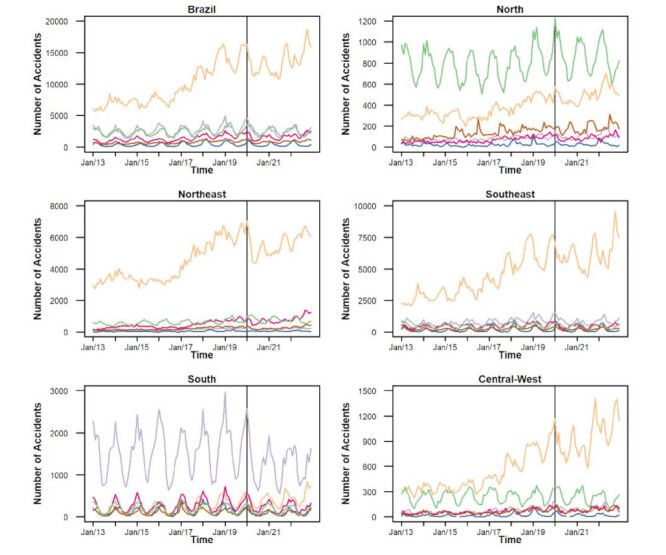
Temporal analysis of the incidence rates of accidents involving venomous animals, by type of accident, in Brazil and the five Brazilian regions between 2013 and 2022.

Figure 3 and Supplementary Tables 1 and 2 describe the temporal analysis of the incidence rates of venomous animal accidents by accident type in Brazil and its five regions from 2013 to 2022. Overall, Brazil showed an increasing trend from 2017 to 2019 (APC: 0.98, p<0.001) and a stable trend from 2020 to 2022 (APC: 0.42, p<0.080). Snakebites exhibited a decreasing trend from 2020 to 2022 (APC: −0.77, p<0.043), while spider bites showed an increasing trend from 2017 to 2019 (APC: 0.39, p<0.002) and a stable trend from 2020 to 2022 (APC: -0.09, p<0.842). Conversely, scorpion stings displayed an increasing trend in all periods: from 2013 to 2016 (APC: 0.046, p<0.001), from 2017 to 2019 (APC: 1.42, p<0.001), and from 2020 to 2022 (APC: 0.63, p<0.004). Bee stings showed two increasing trends: one from 2017 to 2019 (APC: 1.20, p<0.001) and the other from 2020 to 2022 (APC: 1.14, p<0.011). The temporal analysis of incidence rates by the type of venomous animal accidents in the five Brazilian regions from 2013 to 2022 is shown in Figure 3 and Supplementary Table 1. The North region showed a decreasing trend from 2013 to 2016 (APC: -0.28, p<0.036), an increasing trend from 2017 to 2019 (APC: 0.68, p<0.001), and a stable trend from 2020 to 2022 (APC: 0.19, p<0.330). Snakebites in the North region exhibited a decreasing trend from 2013 to 2016 (APC: -0.50, p<0.012) and a stable trend from 2017 to 2019 (APC: -0.52, p<0.089). Conversely, scorpion stings in the North region displayed two increasing trends: one from 2017 to 2019 (APC: 1.12, p<0.001) and the other from 2020 to 2022 (APC: 0.76, p<0.001). Bee stings also showed two increasing trends in the North region: one from 2017 to 2019 (APC: 1.68, p<0.001) and the other from 2020 to 2022 (APC: 1.33, p<0.004) (Figure 3 and Supplementary Table 1). The Northeast region exhibited two increasing trends in venomous animal accidents: one from 2017 to 2019 (APC: 1.25, p<0.001) and the other from 2020 to 2022 (APC: 0.65, p<0.001). Scorpion stings in the Northeast region displayed two increasing trends: one from 2017 to 2019 (APC: 1.20, p<0.001) and the other from 2020 to 2022 (APC: 0.60, p<0.002). Snakebites showed an increasing trend from 2020 to 2022 (APC: 0.40, p<0.001), as did caterpillar stings (APC: 0.91, p<0.001) (Figure 3 and Supplementary Table 1). The Southeast region exhibited two increasing trends in venomous animal accidents: one from 2013 to 2016 (APC: 0.42, p<0.016) and the other from 2017 to 2019 (APC: 1.10, p<0.001), and stability from 2020 to 2022 (APC: 0.26, p<0.312). Scorpion stings displayed an increasing trend from 2017 to 2019 (APC: 1.37, p<0.001) and a stable trend from 2020 to 2022 (APC: 0.51, p<0.070). Bee stings also showed an increasing trend from 2017 to 2019 (APC: 0.68, p<0.001) and a stable trend from 2020 to 2022 (APC: 0.59, p<0.293). The South region exhibited two stable trends: one from 2017 to 2019 (APC: -0.34, p<0.001) and the other from 2020 to 2022 (APC: 0.04, p<0.953).

**Figure 4:**
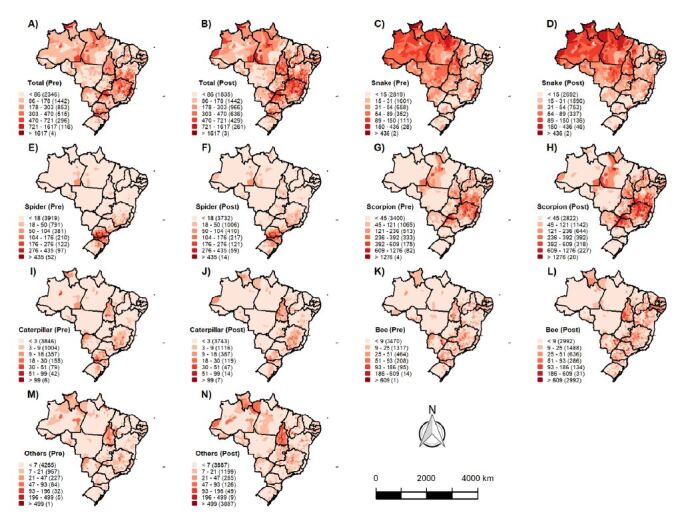
Spatial distribution of the incidence rates of accidents caused by venomous animals in Brazil in the period before and after the COVID-19 pandemic per 100,000 inhabitants. (A) and (B) Total spatial distribution of the incidence rates of accidents involving venomous animals in Brazil in the period before and after the pandemic. (C) and (D) Spatial distribution of the incidence rates of accidents caused by snakes in Brazil in the period before and after the pandemic. (E) and (F) Spatial distribution of the incidence rates of accidents caused by spiders in Brazil in the period before and after the pandemic. (G) and (H) Spatial distribution of the incidence rates of accidents caused by scorpions in Brazil in the period before and after the pandemic. (I) and (J) Spatial distribution of the incidence rates of accidents caused by caterpillars in Brazil in the period before and after the pandemic. (K) and (L) Spatial distribution of the incidence rates of accidents caused by bees in Brazil in the period before and after the pandemic. (M) and (N) Spatial distribution of the incidence rates of accidents caused by other types of venomous animals in the period before and after the pandemic.

Figure 4 illustrates the spatial distribution of venomous animal accident incidence in Brazil and its five regions during the pre- and COVID-19 pandemic periods per 100,000 inhabitants. There was variation in the number of cases per municipality during the pre-pandemic period, with 2,346 municipalities having rates below 86 cases per 100,000 inhabitants, 1,442 municipalities with rates between 86 and 168 cases per 100,000 inhabitants, and 3 municipalities with rates higher than 1,617 cases per 100,000 inhabitants.

**Figure 5:**
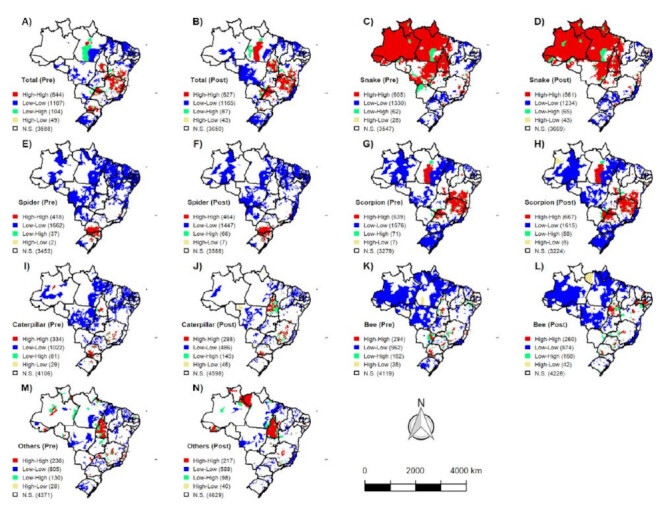
Spatial distribution of the incidence of accidents caused by venomous animals in Brazil and in the five Brazilian regions in the period before and after the COVID-19 pandemic, using the Moran Map. (A) and (B) Total spatial distribution of accidents involving venomous animals in Brazil in the period before and after the pandemic. (C) and (D) Spatial distribution of accidents caused by snakes in Brazil in the period before and after the pandemic. (E) and (F) Spatial distribution of accidents caused by spiders in Brazil in the period before and after the pandemic. (G) and (H) Spatial distribution of accidents caused by scorpions in Brazil in the period before and after the pandemic. (I) and (J) Spatial distribution of accidents caused by caterpillars in Brazil in the period before and after the pandemic. (K) and (L) Spatial distribution of accidents caused by bees in Brazil in the period before and after the pandemic. (M) and (N) Spatial distribution of accidents caused by other types of venomous animals in the period before and after the pandemic.

Figure 5 displays the spatial distribution of venomous animal accident incidence in Brazil and its five regions during the pre- and COVID-19 pandemic periods using the Moran Map. Spatial dependence of smoothed rates was observed both before (Moran’s I: 0.47; p=0.000) and after the COVID-19 pandemic (Moran’s I: 0.50; p=0.000). In total, 644 municipalities exhibited high incidence rates and were considered high-risk areas for venomous animal accidents before the pandemic (Q1 Moran Map). The regions with the highest incidence of accidents during the pre-pandemic period were the Southeast (n=359 municipalities), South (n=185 municipalities), Northeast (n=73 municipalities), North (n=18 municipalities), and Central-West (n=9 municipalities).

**Figure 6:**
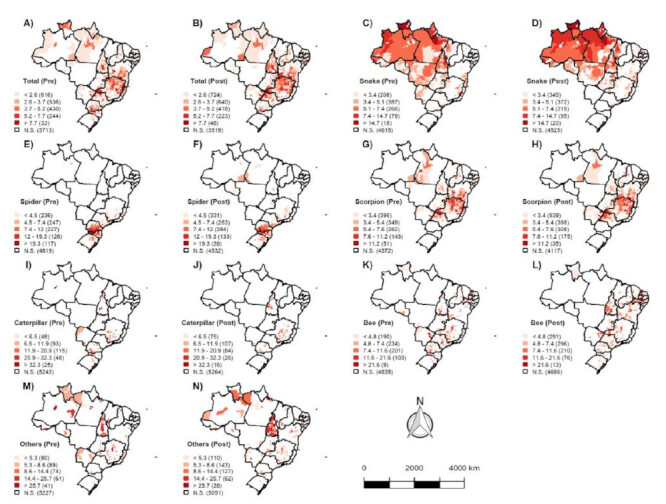
Relative risk of accidents caused by venomous animals in Brazil in the period before and after the COVID-19 pandemic. (A) and (B) Total relative risk of accidents with venomous animals in Brazil in the pre- and pandemic periods. (C) and (D) Relative risk of accidents caused by snakes in Brazil in the pre- and pandemic periods. (E) and (F) Relative risk of accidents caused by spiders in Brazil in the pre- and pandemic periods. (G) and H) Relative risk of accidents caused by scorpions in Brazil in the pre- and pandemic periods. (I) and (J) Relative risk of accidents caused by caterpillars in Brazil in the pre- and pandemic periods. (K) and (L) Relative risk of accidents caused by bees in Brazil in the pre- and pandemic periods. (M) and (N) Relative risk of accidents caused by other types of venomous animals in the pre- and pandemic periods.

Spatial scan statistics identified spatial clusters of high-risk venomous animal accidents in various regions of Brazil, totaling 33 clusters: North (n=13), Southeast (n=13), Northeast (n=8), and Central-West (n=1) (Figure 6).

In the pandemic period, a total of 627 municipalities exhibited high incidence rates and were considered high-risk areas for venomous animal accidents (Q1 Moran Map). The regions with the highest incidence rates of accidents during the pandemic period were the Southeast (n=441 municipalities), Northeast (n=78 municipalities), North (n=43 municipalities), South (n=42 municipalities), and Midwest (n=23 municipalities). A total of 48 municipalities had an RR>7.7 and were in the following regions: Southeast (n=33), Northeast (n=9), Midwest (n=4), South (n=1), and North (n=1) (Figure 6).

Spatial dependence of smoothed rates for snakebite accidents was observed in both the pre-pandemic (Moran’s I: 0.56; p<0.001) and pandemic periods (Moran’s I: 0.50; p<0.001). In the pre-pandemic period, a total of 18 municipalities exhibited an RR>14.7, while in the pandemic period, 20 municipalities showed an RR>14.7. In both periods, municipalities with the highest RR were in the North region. Spatial dependence was also observed for spider bite accidents in the pre-pandemic (Moran’s I: 0.62; p<0.001) and pandemic periods (Moran’s I: 0.56; p<0.001). In the pre-pandemic period, 117 municipalities had an RR>19.3, all of which were in the South region. In the pandemic period, 39 municipalities showed an RR>14.7, located in the South (n=38) and Southeast (n=1) regions (Figures 5 and 6).

Spatial dependence was also observed for scorpion sting accidents in both the pre-pandemic (Moran’s I: 0.58; p<0.001) and pandemic periods (Moran’s I: 0.60; p<0.001). In the pre-pandemic period, a total of 51 municipalities had an RR>11.2, located in the Southeast (n=39), Northeast (n=9), Central-West (n=2), and South (n=1) regions. In the pandemic period, 35 municipalities exhibited an RR>11.2, located in the Southeast (n=22), Northeast (n=8), and Central-West (n=5) regions (Figures 5 and 6).

There was spatial dependence of smoothed rates for caterpillar sting accidents before (Moran’s I: 0.40; p=0.000) and after the COVID-19 pandemic (Moran’s I: 0.25; p<0.001). In the pre-pandemic period, a total of 25 municipalities had an RR>32.3, located in the South (n=10), Southeast (n=10), and North (n=5) regions. In the pandemic period, 16 municipalities exhibited an RR>32.3, located in the Southeast (n=15) and South regions (n=1) (Figures 5 and 6). Spatial dependence of smoothed rates for bee sting accidents was observed before (Moran’s I: 0.19; p<0.001) and after the COVID-19 pandemic (Moran’s I: 0.22; p<0.001). In the pre-pandemic period, a total of nine municipalities exhibited an RR>21.6, located in the Northeast (n=4), Central-West (n=2), Southeast (n=2), and South (n=1) regions. In the pandemic period, 13 municipalities showed an RR>21.6, located in the Northeast (n=11) and South regions (n=2) (Figures 5 and 6).

## DISCUSSION

Our results showed fluctuations in the average incidence rates across all five Brazilian regions in the pre- and COVID-19 pandemic periods. Our analyses revealed temporal stable trends in venomous animal accidents from 2020 to 2022 in Brazil, corresponding to the COVID-19 pandemic period. However, when analyzing accidents by the type of venomous animal across the five Brazilian regions, we observed increasing, decreasing, and stable trends in different periods and regions. The spatial distribution of cases was heterogeneous for both the studied periods, pre- and COVID-19 pandemic. The Southeast and Northeast regions showed a higher RR for scorpion accidents in the pre- and COVID-19 pandemic periods when compared to other regions, while the North region showed a higher RR for snake accidents in both the studied periods when compared to other regions.

During the period from 2020 to 2022, Brazil exhibited a stable trend in the notifications of venomous animal accidents, unlike the period from 2017 to 2019, during which venomous animal accidents showed a growth trend. Research has shown a reduction in the notification of new cases of individuals affected by NTDs during the COVID-19 pandemic^17^. This reduction or stagnation in case notifications, as reported in our study, may be linked to the prioritization of addressing and controlling the COVID-19 pandemic, where the entire healthcare system was mobilized to meet the demands of the pandemic, and consequently, seasonal diseases or conditions took a back seat and ceased to be properly reported^18^. The COVID-19 pandemic, in addition to burdening healthcare systems, exposed healthcare and epidemiological surveillance teams to resource shortages and the limits of physical, psychological, and social exhaustion. Therefore, many cases related to the notification of venomous animal accidents may have been underreported due to the demands related to COVID-19 control^19^.

Between 2020 and 2022, the North, Southeast, and South regions exhibited a stable trend in the notifications of venomous animal accidents, while the Northeast and Central-West regions showed increasing trends. These data contrast with the literature, especially if we consider the period prior to the pandemic (2007-2019), when the temporal trend for most venomous animal accidents was increasing and heterogeneous^20^. The stable trend in the North, Southeast, and South regions is multifaceted and can be explained by the reorganization and improvements in the notification system by the professionals responsible as well as by measures related to awareness, promotion, and implementation based on environmental education^21^. On the other hand, the increasing trend in the Northeast and Central-West regions is uniform and can be explained by the more intense environmental modification and geographic transformation generated in these areas by humans in recent years. The disordered growth of large urban centers, construction of highways, and increased impact of agribusiness are some examples of changes that have reshaped the productive and reproductive food chain, habitat, and migration of venomous animals, thereby contributing to increasingly upward trends^20^.

Our results showed that both in the pre- and pandemic periods, scorpion accidents had a higher incidence and RR in the Northeast and Southeast regions. Studies indicate that scorpions are opportunistic species capable of adapting to environments modified by human actions, including communities with high population density, such as large urban centers^8^. This is a point worthy of reflection, as scorpion accidents exhibit a more urban nature^19^, which may have contributed to the increase in notifications from 2013 to 2022.

In both the pre- and pandemic periods, snakebite accidents had the highest incidence and the greatest RR in the Northern region. Studies indicate that the high level of snakebite incidents in the Northern region is linked to high temperatures and rainfall variations, which trigger increased snake activities in search of food, mating, temperature regulation, and nesting periods^22^ ^,^ ^23^. Additionally, in this region, the main economic activities include agriculture, non-timber extraction, and livestock farming, particularly during rainy seasons, leading to an increase in the number of incidents as farmers become more active in their plantations and animal husbandry activities^26^ ^,^ ^27^.

Spider and caterpillar accidents showed the highest incidence and the greatest RR in the Southern region, in both the pre-pandemic and pandemic periods. Our findings are consistent with other studies^4^ ^,^ ^20^, in which the highest incidence of spider and caterpillar accidents are reported to have occurred in the Southern region. In Brazil, three genera of spiders are of public health importance (*Loxosceles*, *Phoneutria*, and *Latrodectus*)^25^. Species of the *Loxosceles* genus are less aggressive and more prevalent in the Southern region of Brazil. These species adapt to the indoor environment, where they are protected from predation and climatic changes.

The actions of the SINAN deserve close consideration given the scope of this article. In the period preceding the pandemic (2010-2019), research indicated shortcomings in the reporting system, with a high number of ignored cases and missing information, which may have resulted in cases being overlooked and underestimated during this period^24^. However, in the later period from 2019 to 2022, there was a gradual improvement in compulsory notification records, although difficulties remain, such as incomplete notification forms and a lack of adequate training and ongoing capacity building for the professionals responsible. This hinders the accurate measurement of accidents caused by venomous animals in Brazil^28^.

This study analyzed the number of accidents involving venomous animals in Brazil reported to SINAN in the pre- and pandemic periods. We also examined the temporal and spatial trends of venomous animal accidents in Brazil in two distinct periods: pre-pandemic (January 2013 to February 2020) and pandemic (March 2020 to December 2022). However, the results presented here should be interpreted with caution as our investigation relied on secondary data, and the underreporting of cases may have resulted in incidence rates lower than the actual values^29^. Additionally, although ecological studies are useful for formulating hypotheses, their main limitation is that they involve making inferences about individuals based on data aggregated from groups^6^.

Our analyses revealed a trend of stagnation in venomous animal accidents in Brazil and its North, Southeast, and South regions from 2020 to 2022, the period corresponding to the COVID-19 pandemic. Therefore, our findings reaffirm the need for periodic epidemiological studies aimed at understanding the temporal and spatial characteristics of venomous animal accidents in Brazil, thereby aiding decision-making and the development of strategies in areas considered priorities.
